# Response to comment on: Gleicher N et al., 2016. Reprod biol endocrinol Sep 5;14(1):54

**DOI:** 10.1186/s12958-017-0240-y

**Published:** 2017-04-05

**Authors:** Ashley W. Tiegs, James A. Grifo, Santiago Munné, David H. McCulloh, Brooke Hodes-Wertz

**Affiliations:** 1 Department of Obstetrics and Gynecology, New York University School of Medicine, 550 First Avenue, NBV 9E2, New York, 10016 NY USA; 2 New York University Fertility Center, New York University School of Medicine, 660 First Avenue, 5th floor, New York, 10016 NY USA; 3 Reprogenetics, New York, USA

**Keywords:** Mosaicism rate, Blastocyst, Euploid, Misdiagnosis

To the Editors,

We read with interest the recent manuscript of Gleicher, et al. regarding their statement that we described a mosaicism rate of 40%; it is presumed that this conclusion was derived from our report that two of five miscarried embryos (of 525 diagnosed euploid single thawed embryo transfers) were misdiagnosed, which was identified upon reanalysis of biopsied specimens [1]. Our manuscript did not intend (nor was it designed) to assess the mosaicism rate of blastocysts diagnosed by aCGH of trophectoderm biopsies.

Data shows that infants born from in vitro fertilization have low mosaicism rates. NGS-diagnosed euploid trophectoderm samples have been reanalyzed in at least 124 embryos showing 100% euploidy concordance [2–4]. Gleicher, et al. excluded our 280 normal deliveries from the denominator in their extrapolation of our data secondary to the possibility that these infants were derived from mosaic embryos [1]. This exclusion is misleading. To assume that 40% of the infants delivered were derived from self-corrected mosaic embryos is equally misleading.

Our data never intended to evaluate mosaicism rates in embryos, and citing our data in such a way misrepresents our findings. We suggest that instead, the authors should perform experiments to determine the rate of mosaicism experimentally. This should potentially include repeat biopsy of embryos at multiple sites, including the inner cell mass, and subsequently analyzing biopsied specimens using a systemic, validated assay. We are aware of several manuscripts in various stages of the peer review process that actually address this specific question and report rates much lower than the rates that the authors inaccurately extrapolate from our data.
