# Erratum to: Comment on: Gleicher N et al.,2016. Reprod biol endocrinol Sep 5;14(1):54

**DOI:** 10.1186/s12958-017-0246-5

**Published:** 2017-04-10

**Authors:** Ashley W. Tiegs, James A. Grifo, Santiago Munné, David H. McCulloh, Brooke Hodes-Wertz

**Affiliations:** 1 Department of Obstetrics and Gynecology, New York University School of Medicine, 550 First Avenue, NBV 9E2, New York, 10016 NY USA; 2 New York University Fertility Center, New York University School of Medicine, 660 First Avenue, 5th floor, New York, 10016 NY USA; 3 Reprogenetics, New York, USA

## Erratum

The original article [1], was published with the incorrect title “Response to comment on: Gleicher N et al., 2016. Reprod biol endocrinol Sep 5;14(1):54”. The title of this article should read “Comment on: Gleicher N et al., 2016. Reprod biol endocrinol Sep 5;14(1):54” and has now also been corrected online.
